# Current issues and considerations about the central role of rehabilitation therapies in the functional recovery of neurological impairments after stroke in adults

**Published:** 2014-09-25

**Authors:** E Moraru, G Onose

**Affiliations:** * Neurological Rehabilitation Clinic HWK I, Bad Zwesten, WWK – Hessen, Germany; ** "Carol Davila" University of Medicine and Pharmacy, Bucharest; "Bagdasar-Arseni" Clinical Emergency Hospital; Neuromuscular Recovery Clinic, Bucharest, Romania

**Keywords:** stroke, botulinum toxin, locomotion, orthosis, serial casting

## Abstract

Abstract

Well-organized acute and intermediate rehabilitation after stroke can provide patients with the best functional results. Several studies led to major changes in recommendations concerning remobilization therapies following stroke. Controlled studies including early mobilization in stands and training with partial body weight support on treadmills and "gait training" systems showed superior results compared to traditional treatment strategies. In case of spasticity and equinovarus and stiff knee pattern following stroke, botulinum neurotoxin A injections and/or casting enable the achievement of adequate alignment of the ankle for stance phase and allow the improvement of joint mobility during swing phase when restricted.

## Introduction

The stroke is one of the main causes of chronic disability and death [**1**]. It represents an emergency because once the lesion has installed there is no efficient therapy which can restore the function of the brain tissue [**1,2**]. The ideal medical therapy is the prevention of stroke. In addition, efforts have been made in order to precociously diagnose the installation of stroke, in order to be able to intervene during the evolution of a vascular thrombosis. This presupposes that the patients’ medical problems are discovered in the stage of asymptomatic atherosclerosis. Consequently, the therapy for the atherothrombotic disease with the affection of the extra- and intracranial vessels can be divided as it follows: stroke therapeutic primary prevention measures, measures of reestablishing the blood flow and stopping the pathological process in case of stoke, physical and neurological post-stroke rehabilitation therapy, measures of prevention of progression and repeating of the stroke (secondary prevention). These ideas have been also included in the Diagnostic and treatment guide for the cerebrovascular diseases, emitted by the Ministry of Health, 2009 [**1,2**]. 

 The factors which influence the seriousness of the stroke are the following: localization, lesion size, lesion type [**1,2**]. The thrombolysis done by administering TPA (tissue plasminogen activator recombinant) for 4,5 hours is a therapeutic option for ischemic stroke, which aims at reducing the disabilities which appear immediately after stroke [**1**]. The concept "time means brains" has appeared as a result of a research which showed that in the stroke in evolution, 1,9 millions of nervous cells die each minute [**1**]. In the case of the stroke of the vertebrobasilar system, an intervention can be made in 6 hours to remove the arterial thrombus (thrombectomy), by applying interventional radiology (minimally invasive technique) [**1**]. 

 Stages of neurological rehabilitation 

 The functions restoration in the first months after a stroke mostly depends on the spontaneous healing, which is dependant on the compensation potential and the spontaneous plasticity of the brain [**3,4**]. 

 The rehabilitation of the vertical position (orthostatism) and the walking must be started as soon as possible. The first mobilization of the patient should take place during the acute therapy of stroke [**2**]. In addition, the main purpose of precocious rehabilitation is cardiovascular rehabilitation training, reeducation of orthostatism, of feet coordination, of waking, of cognitive functions. Stroke rehabilitation is a process beginning during acute hospitalization and continues with later phases of rehabilitation. The important phases of stroke recovery are acute, subacute and chronic phases (more than 6 months post-stroke) [**2**].

 For documentation of different neurological deficits and of severity of physical disability the best validated assessment instruments are the Barthel Index, Rankin Scale, Scales Ashworth (AS) and Ashworth modified (MAS) for spasticity, Mini Mental State Examination (MMSE) [**2**].

 Locomotion rehabilitation 

 The locomotion therapy mainly encompasses the rehabilitation of orthostatism and walking ability. The walking therapy is realized by an active training of walking which presupposes a frequent repetition. The training oriented towards "functional movements" used to ensure daily basic necessities has proved to be the most effective of all. Movements are "functional" when they allow us to reach an efficient, safe, adapted occupational behavior. The normal movement is possible due to the interaction between the musculoskeletal system and the central nervous system [**1,2,4**]. 

 Researches regarding the physiology of walking have shown that the spinal locomotion centers will be adequately stimulated only at a certain frequency of the steps; this way, a physiologic walking can appear [**5**]. Different authors have stated that the critical speed (the walking cadency) is of 110 steps per minute [**5,6**]. A severely affected patient can reach a number of 50 to 100 steps only if helped by 2 therapists. The training of walking on a walking tape with partial support of the weight with one or two therapists can reach the level of 300 to 400 steps per training. In case of a training supported by robotic equipment and with the help of a therapist, the patient can reach up to 800-1000 steps per therapeutic session. In case of an intense taking over of the walking activities by the equipment used, the rehabilitation process can be slowed because no exercises which involve the use of the patient’s potential are practiced anymore. When referring to walking tape exercises, the degree of difficulty can be progressively raised by establishing new tasks (for example walking with a rucksack, an activity inspired from daily life) [**5,7**]. 

 The robotic locomotion training with the support of the lokomat can accomplish an intensive functional training (a pattern of physiological computer controlled walking), with the possibility of changing the training parameters according to the kinetic object established, as well as the use of a biofeedback and visual feedback of the results [**8,9**]. 

 The application of the things learnt while walking follows after the training supported by the robotic equipment. The other purposes of this training are the following: the raise in the duration and speed of walking, the use of the escalator. The therapist will apply the training of walking outside the training room, as for example in the street, while trying different means of support (from a wheelchair to a walking stick with 1, 3 or 4 sustaining points) which compensate the neurological deficits and can ensure the independence of the patient [**10**]. 

 Orthoses

 The most commonly prescribed orthosis with a view to improving gate is an ankle-foot orthosis, but there are also orthoses for toes, knees, arms, elbow, fist/ hand or/and fingers. The orthosis for the ankle is recommended when the paresis of the dorsal flexion is present or when the spasticity of the flexors is important. In patients with foot inversion due to spasticity, the use of the orthosis allows the improvement in the symmetry of walking and can reduce the consecutive danger of a traumatism [**11**]. 

The orthosis for the knee is most often applied in the precocious rehabilitation stage of walking. In case of knee extensors paresis, it helps in the knee joint stabilization. The knee hyperextension is dangerous in the spasticity of the extensors; the retroversion of the knee can lead to painful joint modifications in time [**4,8**].

 Functional electrical stimulation 

 FES (functional electrical stimulation) uses its effects on the intact neurons and incorporates the movement produced in a functional activity. FES has been used to help the weak or paralyzed muscles develop activities such as orthostatism, walking or movements of the arms, with the support of neuroprosthesis [12-14]. There are developed multifunctional advanced functional electrical stimulation systems that send low-level electrical impulses and can assist patients' functional movement, for example the Neural Electrical Stimulation System (NESS): H(and) 200 și L(imb) 300, 300 Plus [**15**].

 During the precocious rehabilitation period, when the patient is still inactive, electrical stimulation can be used together with the physical exercises in order to maintain the muscular integrity. During the training of walking, it can be directly used the tibialis anterior muscles or the peroneal stimulation nerves at the level o the fibula end, which can lead to the improvement of the dorsal flexion function. The studies show an improved walking condition from the qualitative point of view. It seems that the use of electrical stimulation improves the post-stroke muscular force, as well as the endurance and the muscle force, if FES is administered together with the resistance opposed to the contraction generated by the electrical stimulation of the affected muscles [**10,12**]. 

 Recovery and serial casting

 Serial casting (SC has been included in the treatment of many conditions of the central nervous system (such as brain injuries, lesions of the spinal cord, stroke and multiple sclerosis [**16**]. Most of the times spasticity compels the muscle to remain in a contracted position which shortens it for long period of times; this way it may lead, also through consecutive muscle retractions, to the deterioration and limitation of functional mobility, the loss of the ability of performing routine activities, sometimes associated with severe pains which amplify the functional limitations, altering, in the same time, the quality of life. The joints of the elbow, of the wrist of the hand (radio-cubital-carpal – RCC), of the toes and ankle are the ones which most often benefit from this treatment [**7,16**]. 

 SC is used together with BT-A local therapy, in stroke patients with leg or arm spastic syndrome. A recovery combination with SC and BT-A application is recommeded in a medium or severe spasticity case. Some results show that the mobilization of the joints which are close to the rigid joint treated with BT-A, highlight supplimentary muscular activity. The contraindications in the use of recovery are the following: acute venous thrombosis, peripheral arterial disease, fixed contractures, unstable fractures, periarticular ossification, gout, cutaneous lesions. The purpose of SC therapy is the following: improvement of the passive movement in muscular contracture, reduction of the muscle tone at the level of the joint of the knee or the ankle [**4,16,19**]. 

 Therapy with botulinum toxin type A 

 Botulinum toxin (BT) has been successfully used since 1989 in the therapy of spasticity of the arm and leg, having great results. The principle of action is the reduction of muscular hyperactivity, which influences in a positive manner both the vicious position of the leg/arm and the pain [**18,19**]. From the results of the studies undergone, it seems that BT has an influence at the level of the extrafusal muscle fibers and also at the intrafusal ones, so that it can realize the modulation of the neural relations at the level of the affected muscles, the result being a reduced spasticity. The clinical effect lasts for 2-6 months, according to the size and function of the injected muscle [**19**]. The improvement of the symptoms is also maintained and improved by the physical therapy. The documentation of spasticity is done according to the muscular tone, frequency of the spasms, muscular force, and global handicap. The objectives of the therapy are defined after the neurological examination, together with the physiotherapist and the patient’s family. The aim of this kind of therapy is the improvement of the functional deficits, facilitation of physiotherapeutic exercises, of rehabilitation and care strategies [**19,20**].

 Opposition involuntary muscular hyperactivity that appears against an exogenic force, does not answer to BT therapy, just like posture apraxia, which sometimes mimics spastic conditions. Contracture can also answer to therapy and most of the times the pain is reduced. BT application is not recommended in the following cases: fixed contractures, bone deformities, patients who undergo an anticoagulant therapy. The botulinum toxin is efficient in the therapy of spasticity in upper neuromotor syndrome both in adults and in children [**20**]. 

 In case spasticity is localized in fewer muscles, the local injections of botulinum toxin together with kineto-physical therapy can lead to substantial improvements. In spasticity, there is a need for a careful selection of the muscles and it is important that the injection of the spastic muscles is done strictly at the intramuscular level [**19,21**].

 BT therapy is indicated in spastic syndroms which have the following characteristics [**17,18**]: 

 - dynamical (not fixed) 

 - especially in muscular hyperativity 

 - relevant for the daily activity 

 The use of BT in children presupposes attention to the following: 

 - the dose for muscles according to the body weight 

 - the whole dose for each patient according to the body weight 

 - the period of time between the injections. 

 What has been carefully paid attention to are the effects of the BT-A combined with FES injections, together with functional exercises on spasticity and the functions of hemiplegic patients. The results show that BT-A injections combined with electrical stimulation (ms. tibialis anterior) for 3 days post-injection therapy can improve the functional capacities and spasticity in treated patients [**12**]. The BT-A and neuromotor electrical stimulation (FES) effects on the spasticity of the plantar flexor and the movement amplitude of the dorsiflexion in children with cerebral palsy, were also examined. The management of spasticity of upper limb with botulinum toxin therapy was examined in multicentric study and the most important goals for the clinical practice were defined [**21**].

 Social and professional rehabilitation 

 An extra element, which is important in motor rehabilitation, is social and professional rehabilitation. This way, the rehabilitation and social reintegration therapy of a stroke patient, can be completed [**4,16**]. 

 In addition, occupational therapy has an important role in the rehabilitation process of a stroke patient [**2**]. Its purposes are the following: skills (motor, communication, interaction), areas of activity (daily activities – DA), education, working, playing, social participation. The performances which are necessary to do some normal activities belong to the motor field (coordination, balance, objects manipulation), sensorial and perception field (hearing, visual, tactile), the field of emotional regulation (anger management, frustration, coping strategies), cognitive field (judgment, selection, organization, creation), communication and social life field (gestures, keeping the distance, initiation and answer). In his rehabilitation activity, the occupational therapist must take into account the internal organization of a person (mind-body, musculoskeletal, cardio-pulmonary, neurological performance), the patient’s habits (lifestyle, routine), will (personal belief, preferences, and personal attractions), and the entire cultural, social, spiritual, personal, time context [**4,16**].
